# Immunogenic evaluation of chimeric recombinant protein *against ETEC, EHEC and Shigella*

**DOI:** 10.22099/mbrc.2017.4081

**Published:** 2017-09

**Authors:** Farzane Khalouie, Seyed Latif Mousavi, Shahram Nazarian, Jafar Amani, Poune Pourfarzam

**Affiliations:** 1 Department of Biology, Faculty of Basic Sciences, Shahed University, Tehran, Iran; 2 Department of Biology, Faculty of Sciences, Imam Hossein University, Tehran, Iran; 3 Applied Microbiology Research Center, Baqiyatallah University of Medical Sciences, Tehran, Iran

**Keywords:** Chimeric vaccine, IpaC, CFaB, Intimin

## Abstract

Diarrheal diseases still remain health problem worldwide and out of many bacteria responsible for, *Shigella* and pathogenic *Escherichia* cause the most diarrheas in the world. *Shigella*cause bacterial dysenteries and shigellosis through invasion where the most effective proteins for pathogenesis is Ipac. Critical virulence protein for ETEC infection is CFA/I with two subunits called cfab and cfae. . Attachment of EHEC is the main step of infection and the protein Intimin plays the key role in this function. Protection against the vast majority of responsible pathogens of diarrheas requires development of the combination vaccine against *Shigella*, ETEC and EHEC. In the present study, a multisubunitprotein (CII) containing immunologically significant parts of CfaB, IpaC and Intimin was designed. The chimeric gene (CII) was codon optimized and analyzed with different bioinformatic servers, then synthesized and expressed in *E. coli*. Mice, Guinea pig and, Caco-2 Cell line were used as challenge models for EHEC, *shigella* and ETEC respectively. The chimeric protein induced significant immune response and therefore could be a suitable vaccine candidate against these three pathogens.

## INTRODUCTION

 Diarrhea is considered as one of the common causes of death among children, particularly under five years old [1]. The most prevalent bacterial agents for enteric disease in all age groups, particularly children, are *Shigella*, enterotoxigenic *E. coli *(ETEC) and *enterohaemorrhagic E. coli *(EHEC) [2, 3]. Although different strategies and various efforts have been paid for the development of vaccines against these pathogens, but there is not yet available licensed vaccine [3]. *Shigella* spp. are the most important cause of acute bloody diarrhea (dysentery) [4-6]. Because of increasing antibiotic resistance for *shigella*, development of a suitable vaccine against this bacterium is worthwhile. Pathogenicity of *Shigella* is mostly attributed to its type 3 secretion systems (T3SS) [6, 7]. Proteins secreted via T3SS divert the host cell processes in favor of the bacteria. The invasion plasmid antigens, IpaA and IpaD are the dominant immunogenic antigens and essential virulence factors of *shigellae *[7]. The first effector protein for *Shigella* invasion of epithelial cells is Invasion plasmid antigen C (IpaC) [8]. which acts as an actin nucleation protein and there by mediating membrane lysis [7]. The C-terminal domain of IpaC is required for *Shigella* invasiveness [9]. EHEC are possibly the most important emerging *diarrheal* pathogens of the past decade [10]. Infection of *E. coli*O157:H7 occurs through colonization at the mucosal sites, escaping the host defense system leading to host cell damage by reproduction. Intimin, the bacterial outer-membrane protein, plays a critical role for development of EHEC infection. It is required for attaching to the host cell,in order to form the attaching effacing lesions (A/E lesions) [11]. Receptor binding activity of the intimin is present at 280 amino acids in the C-terminal (Int280) which mediates the interaction with translocated intimin receptor (Tir) [12, 13]. Int280 could induce strong immune response in serum and colostrums of pregnant swines [14]. Enterotoxigenic *Escherichia coli* (ETEC) is being one of the main cause of diarrhea among children and travelers [15, 16]. Attachment of ETEC to epithelial cells of the small intestine occurs by means of colonization factors (CFs). After attachment, bacteria produce toxins in the vicinity of the intestinal epithelium where it causes watery diarrhea. Colonization factor antigen I (CFA/I) is the most important between epidemiologically relevant CFs. The major structural and minor tip subunits of CFA/I are CfaB and CfaE, respectively.[17-19]. Development of polyvalent vaccines can reduce the cost effect and frequency of vaccine administration [20]. In order to reach for a efficacious combination vaccines for the prevention of infections caused by ETEC,EHEC and Shigella, in the present research a new structural model consisting of whole Cfab, 282 amino acids from the C-terminal of Intimin, and Ipac64 (residues 300-363 of this protein) were designed with bioinformatic tools. An silico approach was used to analyze the structure, stability and immunogenic potentiality of the designed chimeric protein. The chimeric gene was synthesized and expressed in *E.coli. *The immunogenic properties of the chimeric protein were examined after its administration to different animal models.

## MATERIALS AND METHODS

**Bacterial strains, plasmids and media:** *S. flexneri*2a, *E. coli*O157:H7, ETEC,* E. coli*BL21 (DE3) and *E. coli* DH5 α were prepared from Shahed University of Iran. Expression vector pET-32a was from Novagen (USA). All bacterial strains were grown in LB broth at 37^◦^C, the medium was supplemented with ampicillin (100μg/mL) whenever required.

**Figure 1:**
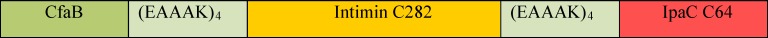
Diagram of constructed recombinant protein CII containing CfaB, Intimin, and IpaC

**Designing and construction of chimeric CII: **The sequences of the gene encoding CfaB, Intimin C282 and IpaC C64 were obtained from GenBank. These sequences were used to generate a trivalent proteins with linkers (EAAAK)_4_ in between [22]. and the restriction sites for enzymes *Eco*RI and *Hind*III at the 5́ and the 3́ respectively (Fig 1). The codon optimization was carried out by the Genscript Optimization Gene ^TM^ algorithm and bioinformatic analysis was performed as explained previously (23). The gene encoding target protein was synthesized by Shine Gene Molecular Biotech, Inc. (Shanghai, China) on pUC57 cloning vector.

The synthetic gene was sub cloned into pET32a with the 6XHis- tag at the N-terminal. The pET-CII plasmid was transformed into *E. coli* strain BL21 (DE3) and cultured in LB medium at 37°C till OD_600_ reached 0.5-0.7. IPTG (BanglorGenai) with the final concentration of 1mM was then added to the bacterial culture and further incubated for 5 hours at 37°C. Cells were harvested by centrifugation at 14000×g/15 min and each pellet was resuspended in 100μl of lysis buffer (1mM EDTA, pH 8.0, 500mMNaCl, 0.12 mg/ml PMSF, 0.3mM Metheamen, 5mM Imidazol, 200mg/30ml MgCl_2_). The cell lysate was analyzed by 10%sodium dodecyl sulfate–polyacrylamide gel electrophoresis (SDS-PAGE).

**Purification of recombinant fusion protein: **Recombinant CII was purified following expression, using nickelchelation affinity chromatography (Ni-NTA). Bacterial pellet from 100 ml culture was thawed, resuspended in 6ml lysis buffer (50mM NaH_2_PO_4_, pH 8.0, 300mM NaCl, 10mM imidazole, 0.2 mg/ml lysozyme) and sonicated for 20 sec pulse and 15 min rest (4times). The lysate was then centrifuged at 14,000×g for 20 min. The supernatant was poured into the Ni–NTA column and washed with denaturing buffers containing 8M urea (100 mM NaH2PO4, 10 mM Tris-HCl, 8 M Urea) and the flow-through of the soluble fractions were collected and analyzed on 12% SDS-PAGE.

**Western blot analysis: **Purified CII was electrophoresed and transferred from SDS-PAGE to nitrocellulose filter using transfer buffer (150mM glycine, 20mM Tris-base and 20% methanol) and Bio-Rad Mini Protean II System. The membrane was soaked in the blocking buffer of 5% milk/phosphate-buffered saline (PBS, 137mMNaCl, 2.7mMKCl, and 4.3mMNa2HPO4, pH7.3) andincubated at 4 ◦C overnight with gentle agitation. The membrane was then incubated in a 1:1000 dilution of mice anti-His-tag IgG in the PBS/T (PBS contain 0.05% Tween 20), with gentle shaking at 37 ◦Cfor 1 h. The membrane was washed with PBS/T three times and incubated in 1:50,000 dilution of HRP-conjugated goat antimouseIgG(Abcam), with gentle shaking at 37 ◦C for 1 h. The filter was washed three times with PBS/T and protein band was detected using substrate solution,3,3’-diaminobenzidine (DAB)containing 1μl/ml H2O2 . Chromogenic reaction was stopped by washing the filter twice with PBS.

**Animal immunization: **Ten female BALB/C mice (Pasteur Institute of Iran) were randomly divided into 2 groups of 5 animals.Animals of the test group were injected subcutaneously with 20μg purifiedCII protein emulsified with complete Freund’s adjuvant (Razi institute). Booster doses of15μg and 10μg CIIwith incomplete Freund’s adjuvant were injectedafter 15 and 30 days respectively. 5μg CII was given intraperitoneally 15 days after the last booster,. PBS was injected throughthe same route to control group animals. Blood samples were collected from the mice one week after the second, third and fourth injections. The sera were collected and stored at −70 ◦C for further analyses.6 female guinea pigs weighing 250to 300 g (Pasteur Institute of Iran) were divided into test and control groups. The test group was immunized subcutaneously with recombinantCIIin a series of four injections starting with 20μg followed by 15, 10 and 5μgat 2-week intervals. PBS was injected to control guinea pigs with the same procedure. Blood samples were collected one week after the second, third and fourth injection for further studies.

**Antibody responses to recombinant CII: **Antibody responses were determined by western blot and enzyme linkedimmunosorbent assay (ELISA).ForELISA, 96-well plates (Caspian) were coated with 5μg of CII protein in coating buffer (64mM Na_2_CO_3_, 136mM NaHCO_3_, pH 9.8) and blocked for overnight at 4 ◦C. The plates were washed three times with phosphate-buffered saline (PBS) containing tween 20(PBS/T) and the non-specific sites were blocked with 5% milk in PBS/T. Mouse serum samples were serially diluted from 1:100 to 1:12800in PBS/T and added to the plates and incubated at 37◦C for 45 min. None immunized mice sera were used as control. After 3 times washing with PBS/T, plates were incubated with1/50,000 dilution of Goat Anti-Mouse HRP (IgG H&L) (ab97023) | Abcam at 37◦C for 30 min and washed three times in PBST. The wells were added with 100μl of citrate buffer containing 0.06% (W/V) of O-phenylenediaminedihydrochloride (OPD) (MERCK) and 0.06% (V/V) hydrogen peroxide and incubated at room temperature for 15 min. The reaction was stopped with 100μl of 2MH_2_SO_4_ and the OD_492 _was read on a microplate reader. Serum IgG antibody from guinea pigs was also estimated with ELISA using guinea pig serum and Goat Anti-Guinea pig HRP (IgG H&L) (ab6908) | Abcam as described above.

 For western blotting, recombinant CII was separated on 12% SDS-PAGE and transferred onto nitrocellulose membranes. The mouse CII antisera were used as the primary antibody at 1:1000 dilutions. HRP-conjugated goat anti-mouse IgG (1:50,000, Abcam) antibody was used as a secondary antibody and the colorimetric reaction was visualized using substrate development buffer (BangalorGenai). 

**Immunized mice challenge: **The mice were challenged two weeks after last immunization. In order to reduce the normal bacterial flora of the gut, mice were drank water containing 5 mg/ml streptomycin sulfate prior to challenge [24]. After overnight fasting, mice were fed with 10^10^ colony forming units (CFU) of E. coli O157:H7 in 100μl of PBS. The fecal samples from each mouse were collected at two days interval for four weeks. E. coli O157:H7 fecal shedding was monitored by adding approximately 0.1 g of feces to 1ml of LB broth followed by incubation at room temperature for 2–4 h to allow the fecal pellets to soften. The mixture was vortex and serial dilutions of the supernatant were plated onto Sorbitol MacConkey agar plates. Plates were incubated overnight at 37◦C and E. coli O157:H7 colonies were counted [25, 26]. The keratocon-junctivitis test was carried out for *shigella* challenge. An overnight LB-culture of S*. flexneri*was centrifuged and the bacterial sediment was resuspended in normal saline. Guinea pigs' eyes were inoculated with 5 x 10^8^ organisms and the animals were inspected for the development of conjunctival infection for 48 h. The disease was classified on the basis of intensity of clinical symptoms [27, 28]. The severity of infection in the eyes was rated after challenge by the following four-point scale: 0= no disease or mild irritation; 1= lacrimation or eyelid edema; 2=keratoconjunctivitis, but no purulence; 3= full purulent keratoconjuctivitis. Score of 0 or 1 was defined as full or partial protection [28, 29].The binding inhibition assay of ETEC in the presence and absence of CII antiserum were determined by performing attachment assays using the Caco-2 human intestinal cell line as described previously [30]. 

To analyze the effect of antibody on bacterial growth, monolayers of Caco-2 cells were prepared in six-well Falcon tissue culture plates. ETEC cells in the exponential growth phase were washed three times with PBS. The immunized mice antisera were diluted in LB broth to a final concentration of 1:250.300μl of bacterial suspension pretreated with 150μl of immunized mice antisera were added to two plates containing Caco-2 cells. The serum from non-immunized mice served as negative control. Plates were incubated for 1h.The plates were trypsinized and bacterial cells attached to caco-2 cell were cultured overnight in CFA agar. Colonies were counted from each sample. Log 10 CFU/ml values were calculated from the mean of triplicate samples and subjected to statistical analyses [31, 32].

**Statistical analysis: **The data are representative of three independent experiments expressed as the mean±standard deviation (SD). All statistical analyses were performed by a SPSS 12.0 statistical program. The data for antibody responses between immunized and non-immunized groups was analyzing using Student t-test. Results for the adhesion inhibition assay were presented as mean percentages of bacterial attachment ± SD. A value of P<0.05 was considered statistically significant.

## RESULTS

**Figure 2:**
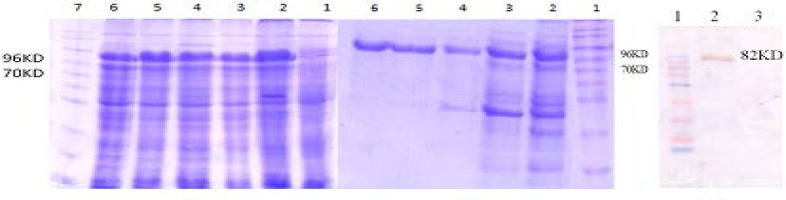
Expression, Western blot analysis and purification of recombinant CII. A) Optimization of recombinant CII (lanes 2-6) with 1mM IPTG. Lane 7, protein weight marker. Lane 1, uninduced E. coli BL21DE3/pET32a + cII gene as control. B) Purification of recombinant CII protein with 6X-His-tagged from pET32.Lane1, protein weight marker. Lane 2, flow-through. Lane 3, wash column with 40mM imidazole, Lanes 4-6, purified protein after elution with 250mM imidazole. C) Western blot analysis of CII using anti 6X-His-tag antibodies. Lane 1, protein weight marker. Lane 2, CII. Lane 3, total protein of E. coli BL21DE3/pET32 without cII gene after induction as control

The chimeric protein was designed and analyzed by bioinformatics’ software as described previously [22]. The gene encoding the chimeric protein was synthesized and subclone in pET32 and transferred to *E. coli *(BL21DE3). The synthetic gene was expressed in E. coli (BL21DE3) (Fig. 2A). The expression of recombinant CII protein was confirmed by Western blotting using the anti-His-tag antibodies (Fig. 2C). Purification of CII was carried out and SDS-PAGE analysis manifested the presence of protein band of about 82 kD where CII with approximate MW of 59 kD is fused to about 22 kD N-Trx tail of pET32 vector( Fig. 2B).

**Figure 3:**
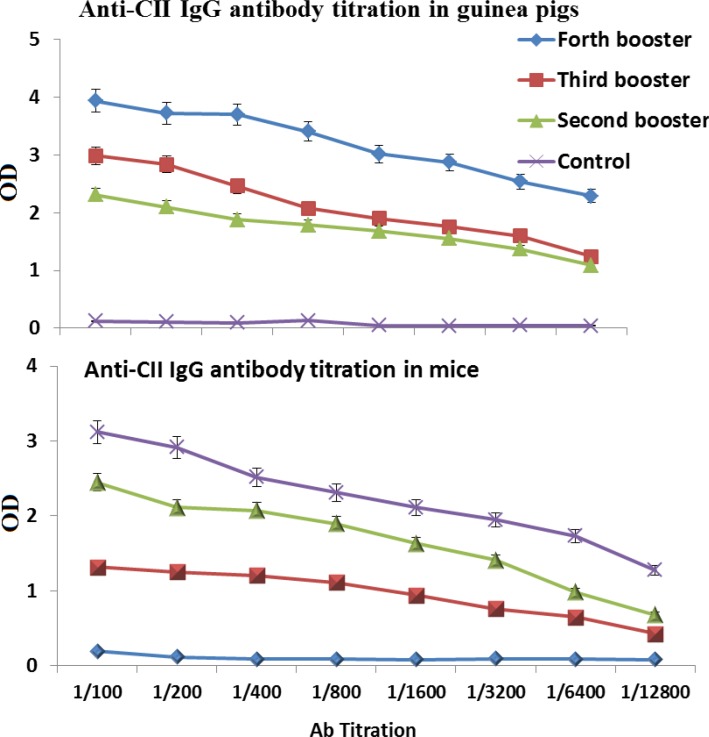
CII -specific serum IgG following subcutaneous immunization. The sera were collected after second immunization and assessed for CII-specific IgG in guinea pigsand in mice by ELISA method. Non-immunized animals sera were used as control

Mice and Guinea pig immunized subcutaneously with purified CII protein. The anti-CII IgG antibody titer in both mice and Guinea pig test groups were significantly (P<0.01) increased compared to control groups. (Fig. 3). The Western blot carried out with anti-CII mice IgG antibody showed that purified antibody specifically binds to recombinant chimeric protein (data not shown).

**Figure 4:**
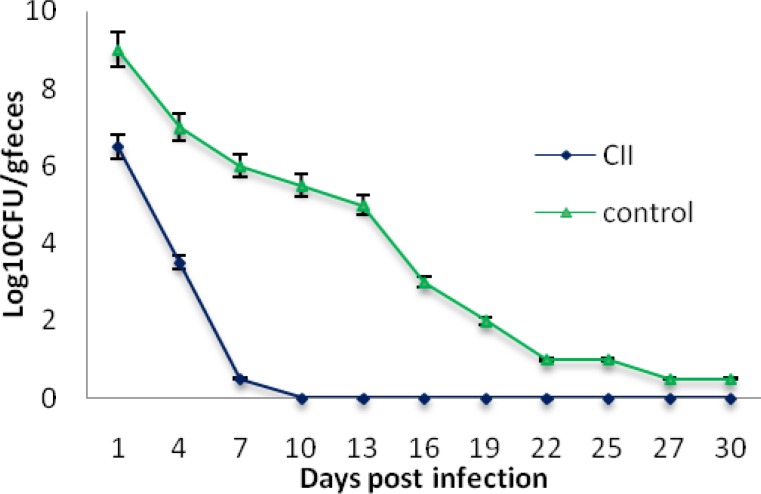
*E. coli* O157:H7 shedding in feces following subcutaneous administration in mice. CII Immunized and non-immunized mice (control) were orally fed 10^10^*E. coli* O157:H7 and shedding was monitored in the feces for four weeks

*E. coli* O157:H7 was fed to immunized and non-immunized control mice and the shedding of EHEC was monitored in feces. Non-immunized control mice shed high levels of *E. coli* O157:H7 in their feces over the four-weeks sampling period whereas that of immunized mice was reduced gradually and stopped completely after ten days (Fig. 4).

**Figure 5:**
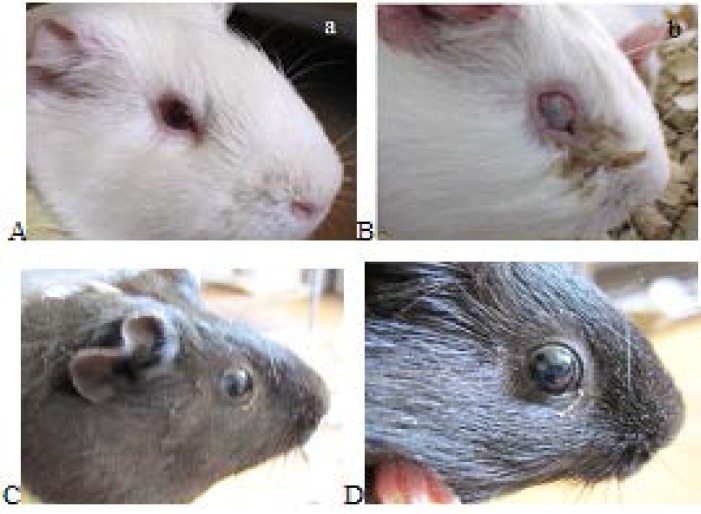
Sereny test with *Shigella* in immunized and nonimmunized guinea pigs . A: control pigs that during the injection alone PBS was injected ,B: purulent infections of eye in control guinea pigs there was after 3 days of instilled *shigella* into their eyes, C:CII immunized guinea pig, D: immunized guinea pig after *Shigella* inoculation and There is no sign of infection

All infected eyes in Non-immunized control guinea pig developed moderate to severe conjunctivitis with hyperemia, edema and discharge, showed full purulent keratoconjuctivitis (score 3).Immunized guinea pig didn’t display any disease and member in score 0 (Fig. 5).

**Figure 6:**
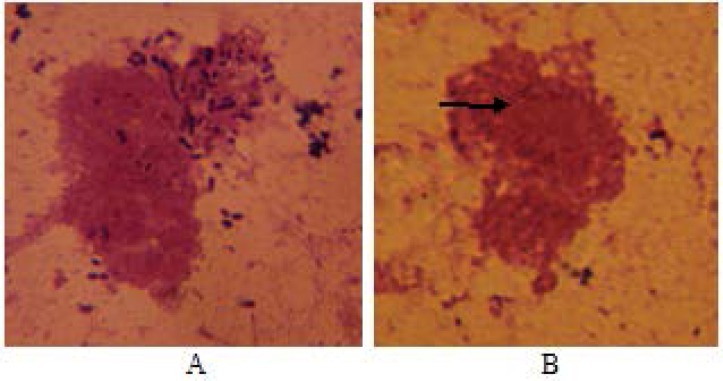
Binding of ETEC strain to Caco-2 cells. Before incubation with Caco-2 cells, the bacterial cells were pretreated with non-immune serum (A) and anti-CII (B

ETEC cells pretreated with serum from the control mice group were attached onto Caco-2 cells, whereas pretreated EHEC cells with immunized mice antisera significantly blocked their binding to Caco-2 cells. 41% of the Caco-2 cells were found to attach the bacterial cells pretreated with immune mice sera against CII (Fig. 6).

## DISCUSSION

Diarrheal diseases are responsible for mortality of about 750 thousands children under 5 years old, particularly in developing countries. ETEC, EHEC and Shigella are among the main causative agents of diarrhea [33]. To our knowledge, a suitable vaccine is not currently available to protect against these three bacteria together. Therefore any vaccine candidate designed to prevent their infection, if collectively contains the major surface antigens of these bacteria, may play a key role in preventing their binding to the colonic epithelial cells. With this hypothesize in our mind; we selected 3 surface antigens (each from one bacterium) which are playing important role in attaching these bacteria to the intestinal epithelium. These three antigens are Intimin, CfaB and IpaC. Intimin is an outer membrane protein required for EHEC attachment to gastrointestinal tract [11, 34-35]. Antibodies raised against intimin prevented the initial steps of bacterial colonization in the the colonic epithelial cells [36, 37]. Carvalho(2005) also demonstrated that the C-terminal of intimin binds to its translocated receptor to cause attaching and effacing lesion during infection(38). Adhesion of the ETEC to the small intestinal epithelium was mediated by colonization factors (CFs). One of the most common CFs are CFA/I with a major subunit, CfaB [39, 40]. Antibody against CFA/I protected Caco-2 cells from bacterial attachment [30]. IpaC has been identified as the first factor for *Shigella *invasion of epithelial cells. The C-terminus of IpaC may possess this function [8, 41]. The chimeric protein CII was designed from antigenic part of these protein linked together with a spacer (EAAAK)4 to prevent their structural interferences analyzed with in silico performance [23]. To confirm this claim, this project was carried out in the laboratory with animal models and CII significantly protected animal models compared to their control groups. The successful experience of using (EAAAK)4 sequences in chimeric genes have paved the way for it to bring about rationally acceptable results [13, 20, 42] . The chimeric protein was injected in four booster doses with gradual decrease of its concentration to raise affinity and thereby fully activate the B cell immune cascade. Finally, last intraperitoneal injection was given to activate B cell with more differentiation effectively [43, 44]. ELISA was used to determine the amounts of antibodies in the serum. There was a significant difference in the antibody titer between sera of the immunized and control groups. Production of specific antibodies was confirmed by Western blotting (Fig. 3). Mice were considered as animal model for EHEC. Immunization with chimeric protein CII strongly protected mice challenged with *E. coli* O157:H7 and immunized mice did not shed EHEC following infection (Fig. 4) indicating that CII-specific antibodies could prevent from EHEC colonization. Although mucosal immunization leading to IgA production has crucial rule in inhibiting colonization of bacteria selected for this research, but our present data and previous reports [13]. indicate that the IgG can pass from epithelial cells and inhibits bacterial attachment to the gut [45]. 

 Guinea pigs were tested for CII protective efficacy and immunogenicity against shigellosis [46]. No sign of keratoconjunctival infection following challenge with a *Shigellaflexneri*2aconferred protection in immunized guinea pig (must be re-writte) (Fig. 5). Anti CII serum could also inhibited ETEC adhesion to Caco-2 cells significantly (Fig. 6).Wang* et al *obtained partial protection using recombinant OmpC of ETEC [47]. Gohar et al immunized mice with a five main diarrheagenic pathotypes of formalin killed *E coli.* The combined vaccine protected animals against all five bacterial strains. Our results are compatible with the results worked on vaccine against all pathotypes of *E.coli. * Although there are reports on chimeric or heat killed cocktail vaccine development against various pathogenic strains [48]. few reports are available on multi species vaccines development. Our findings could shed a light on this issue. 
